# Synchronous gastrointestinal cancer and gastrointestinal stromal tumors: a single-institution experience

**DOI:** 10.1186/s12957-016-0882-9

**Published:** 2016-04-29

**Authors:** Jian Du, Ning Shen, Hai-Shan He, Xiao-Lan Fu, Jing-Zhong Wang, Chong-Zhou Mao

**Affiliations:** Department of General Surgery, Suining Municipal Hospital of Traditional Chinese Medicine, Suining, 629000 Sichuan China

**Keywords:** Gastrointestinal stromal tumors, Synchronous, Clinicopathological, Prognosis, Digestive malignant tumors

## Abstract

**Background:**

A study was conducted to investigate the clinicopathological features and survival outcomes of gastrointestinal stromal tumors (GISTs) that are synchronous with other gastrointestinal cancers.

**Methods:**

Clinical and pathological data of 286 patients with primary GIST from a single institution from January 2009 to December 2014 were reviewed.

**Results:**

The entire study population comprised 286 patients with GISTs. Of these patients, 167 (58.4 %) were males and 119 (41.6 %) were females. The median age was 58 years old (in the range 29–86 years). A total of 47 patients were diagnosed with GISTs synchronous with other digestive tract malignancies (synchronous group), whereas 239 patients were diagnosed with non-synchronous disease (non-synchronous group). The concomitant digestive tumors in 27, 12, 7, and 1 patients were diagnosed as gastric carcinoma, esophageal carcinoma, colorectal carcinoma, and pancreatic adenocarcinoma, respectively. Compared with the synchronous group, the non-synchronous group exhibited a higher percentage of increased mitotic count (*P* = 0.011). The difference in tumor diameter between the two groups was statistically significant (*P* < 0.001). Patients in the non-synchronous group exhibited larger tumor size than the patients in the synchronous group (5.9 ± 3.5 cm vs. 1.6 ± 0.4 cm, *P* < 0.001). The majority of GIST lesions in the synchronous group were located in the stomach (*P* = 0.020). Lower risk stratifications and worse ECOG performance statuses were observed in the synchronous group (*P* < 0.001) than in the non-synchronous group. The 5-year overall survival rate was significantly higher in patients with no synchronous digestive tract malignancies than in patients with synchronous disease (70.8 vs. 34.1 %, *P* < 0.001).

**Conclusions:**

Patients with GIST synchronous with other gastrointestinal cancers show worse prognosis than those with non-synchronous tumors. Clinicians should pay more attention to this subgroup.

## Background

Gastrointestinal stromal tumors (GISTs) are the most common type of mesenchymal tumors in the gastrointestinal tract [1]. The incidence of GISTs in China has increased in recent years [2]. Most GISTs are diagnosed incidentally during investigative or therapeutic procedures for unrelated diseases. The ratio of patients with GIST that is diagnosed to be synchronous with another digestive neoplasm is reportedly in the range of 17.1 to 37.9 % [3–5]. Numerous patients with GISTs that are synchronous with other neoplasms have been described previously, but most of these cases were published in case reports or are included in studies with small sample sizes.

Although major advances in the management and molecular biology of GISTs have been achieved in the past two decades, little is still known about GISTs coexisting with gastrointestinal tumors. Furthermore, the effect of the coexistence of GISTs and other primary gastrointestinal neoplasm on patients remains controversial [6, 7]. Familiarity with occurrence patterns of GISTs synchronous with other neoplasms is important for both pathologists and surgeons. In the present study, we analyzed the clinicopathological characteristics and treatments of a large sample size of patients with GISTs synchronous with other gastrointestinal malignancies from a single institute.

## Methods

### Patient selection

Patients with gastrointestinal tumor synchronous with other digestive malignancies were identified by clinical data at the Department of General Surgery, Suining Municipal Hospital of Traditional Chinese Medicine from January 2009 to December 2014. All primary GISTs were resected and histologically diagnosed by a pathologist. Patients with GISTs diagnosed as recurrent or metastatic and those with malignancies other than malignant digestive tumors with GISTs were excluded. The Institutional Review Board and Ethics Committee of the Suining Municipal Hospital of Traditional Chinese Medicine deemed that an ethical review was unnecessary for this retrospective study.

### Data collection and follow-up

Data on age at diagnosis, gender, size, and tumor location, Eastern Cooperative Oncology Group (ECOG) performance status, medication, surgical outcome, mitotic count, and survival outcome of the patients were collected. The risk stratification of GISTs was evaluated according to the modified National Institutes of Health classification [8]. Surgery performed for the management of the tumors was classified into three categories, as follows: R0 (complete gross and microscopic resection), R1 (with microscopic residual lesions), and R2 resections (with retention of any gross residual tumors). Follow-up was conducted by telephone call, office visit, or outpatient clinic visit from February 2015 to May 2015. Abdominal CT, blood routine examination, and evaluation of liver and kidney functions were performed.

### Statistical analysis

Overall survival (OS) was defined as the duration from the start of treatment until death from any cause or the last follow-up visit. All statistical analyses were performed using the Statistical Package for Social Sciences (SPSS Inc., Chicago, IL, USA). The differences between groups were analyzed using ANOVA for continuous variables and *χ*^2^ test or Fisher’s exact test for categorical data. Measurement data were expressed as mean ± standard deviation. Survival analysis was performed using the Kaplan-Meier method and the results were compared using a log-rank test. Differences with two-sided *P* < 0.05 were considered statistically significant.

## Results

### Patients’ characteristics

**Fig. 1:**
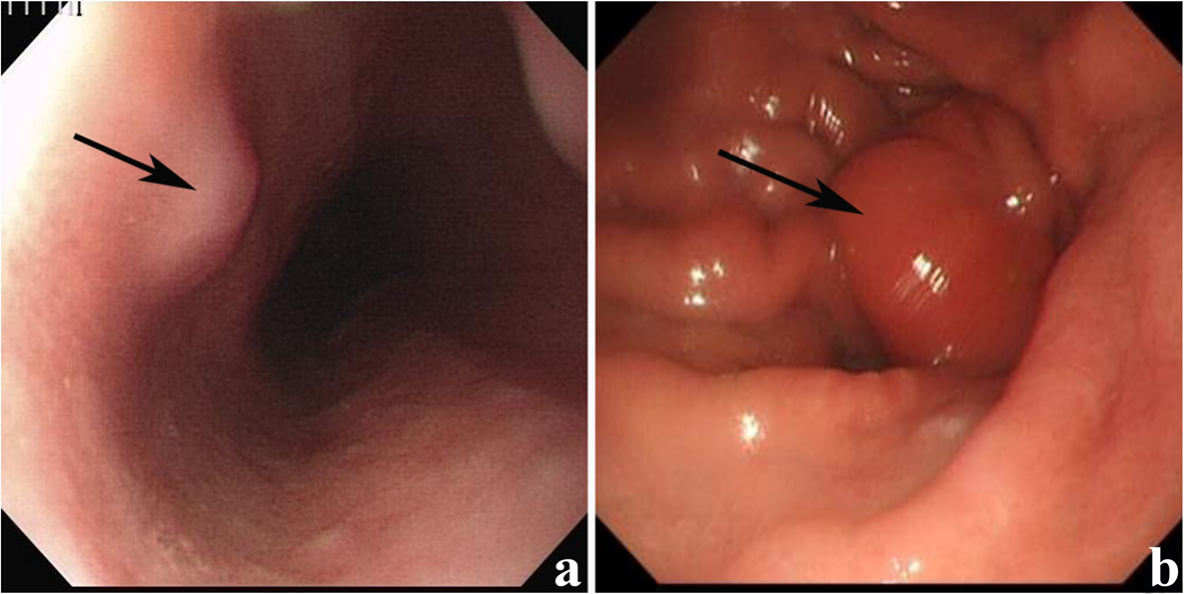
**a**, **b** Electronic endoscopy image showing tumors located in the stomach (**a**) and the large intestine (**b**)

The entire study population comprised 286 patients with GISTs, including 167 (58.4 %) males and 119 (41.6 %) females. The median age was 58 years old (29–86 years old). Table 1 summarizes the baseline characteristics of the GIST patients. Of the 286 patients enrolled, 47 patients were diagnosed with GISTs that are synchronous with other digestive tract malignancies (synchronous group), whereas 239 patients exhibited no synchronicity (non-synchronous group). The number of cases with tumors located in the stomach, small intestine, and other parts (omentum, retroperitoneum, mesentery of the large and small intestine, and pelvis) were 219 (76.6 %), 52 (18.2 %), and 15 (5.2 %), respectively. In the synchronous group, the tumors in 2 out of 47 patients were preoperatively discovered by electronic endoscopy and subsequently diagnosed as GISTs; Fig. 1a, b). The data are shown in Table 1. Among the patients, 15 and 141 received preoperative imatinib mesylate (IM) and adjuvant IM therapy, respectively. A total of 31 patients had tumor metastasis at the time of diagnosis or during surgery. R0 resection was completed in 274 patients (95.8 %).

**Table 1:** The demographic and tumor characteristics of patients with GISTs

Variables	No. of patients (*n* = 286)	Percentage (%)
Age (years)		
≤ 60	165	57.7
> 60	121	42.3
Gender		
Male	167	58.4
Female	119	41.6
ECOG score		
≤ 1	190	66.4
≥ 2	96	33.6
Tumor size (cm)		
≤ 5	127	44.4
> 5	159	55.6
Tumor location		
Stomach	219	76.6
Small intestine	52	18.2
Others^a^	15	5.2
Mitotic count (50 HPF)		
≤ 5	173	60.5
6–10	86	30.1
> 10	27	9.4
NIH risk categories		
Very low	56	19.6
Low	62	21.7
Intermediate	93	32.5
High	75	26.2
Surgical margins status		
R0	274	95.8
R1/R2	12	4.2
Synchronous with digestive malignancies	47	16.4
Preoperative IM therapy	15	5.2
Adjuvant IM therapy	141	49.3
Metastasis at diagnosis or surgery	31	10.8
Median follow-up (range, months)	32 (5–76)	-
Hospital stay (days, mean ± SD)	17.3 ± 4.5	-

*GISTs* gastrointestinal stromal tumors, *NIH* National Institutes of Health, *HPF* high power fields, *IM* imatinib mesylate, *SD* standard deviation

^a^Including omentum, retroperitoneal, mesentery of large and small intestine, and pelvic mass

### Tumor characteristics

A total of 47 patients were diagnosed with GISTs that are synchronous with other digestive tract malignancies. The concomitant digestive tumors in 27 (57.4 %), 12 (25.5 %), 7 (14.9 %), and 1 (2.1 %) patients were diagnosed as gastric carcinoma, esophageal carcinoma, colorectal carcinoma, and pancreatic adenocarcinoma, respectively. For the patients with gastric carcinoma, TNM staging were as follows: I in 4, II in 7, III in 15, and IV in 1. For the patients with esophageal carcinoma, 1 case was diagnosed as stage I, 5 as stage II, and 6 as stage III. Compared with the synchronous group, the non-synchronous group showed a higher percentage with increased mitotic counts (*P* = 0.011). The difference in tumor diameter between the two groups was statistically significant (*P* < 0.001). Patients in the non-synchronous group exhibited larger tumor sizes than those in the synchronous group (5.9 ± 3.5 cm vs. 1.6 ± 0.4 cm, *P* < 0.001). The majority of the GIST lesions in the synchronous group were located in the stomach (*P* = 0.028). Lower risk stratifications and worse ECOG performance statuses were observed in the synchronous group than in the non-synchronous group (*P* < 0.001). Meanwhile, no statistically significant difference in age, gender, and hospital stay was noted between the two groups (Table 2). Overall, 53 GISTs were found in the synchronous group due to multiple GISTs which were detected in 5 patients.

**Table 2:** Demographic and clinicopathologic data between two groups

Variables	Synchronous group (*n* = 47, %)	Non-synchronous group (*n* = 239, %)	*P* value
Age, years	66.4 ± 5.6	61.2 ± 4.2	0.231
Gender			0.614
Male	29 (61.7)	138 (57.7)	
Female	18 (38.3)	101 (42.3)	
ECOG score			<0.001
≤ 1	16 (34.0)	174 (72.8)	
≥ 2	31 (66.0)	65 (27.2)	
Tumor site			0.028
Stomach	43 (91.5)	176 (73.6)	
Small intestine	3 (6.4)	48 (20.1)	
Others^a^	1 (2.1)	15 (6.3)	
Tumor size, cm	1.6 ± 0.4	5.9 ± 3.5	<0.001
Median (range, cm)	0.7 (0.2~2.5)	5.25 (1.5~30.0)	
Mitotic count (50 HPF)			0.011
≥ 10	0 (0.0)	27 (11.3)	
< 10	47 (100.0)	212 (88.7	
NIH risk classification			<0.001
Very low and low	42 (89.4)	76 (31.8)	
Intermediate and high	5 (10.6)	163 (68.2)	
IM adjuvant treatment			<0.001
Yes	2 (4.3)	139 (58.2)	
No	45 (95.7)	100 (41.8)	
Synchronous cancer			
Esophageal carcinoma	12 (25.5)	–	
Gastric carcinoma	27 (57.4)	–	
Pancreatic adenocarcinoma	1 (2.1)	–	
Colorectal carcinoma	7 (14.9)	–	
Hospital stay, days	18.6 ± 5.3	16.9 ± 4.2	0.209

*ECOG* Eastern Cooperative Oncology Group, *HPF* high power field, *NIH* National Institutes of Health, *IM* imatinib mesylate

^a^Including omentum, retroperitoneal, mesentery of large and small intestine, and pelvic mass

### Survival outcomes

**Fig. 2:**
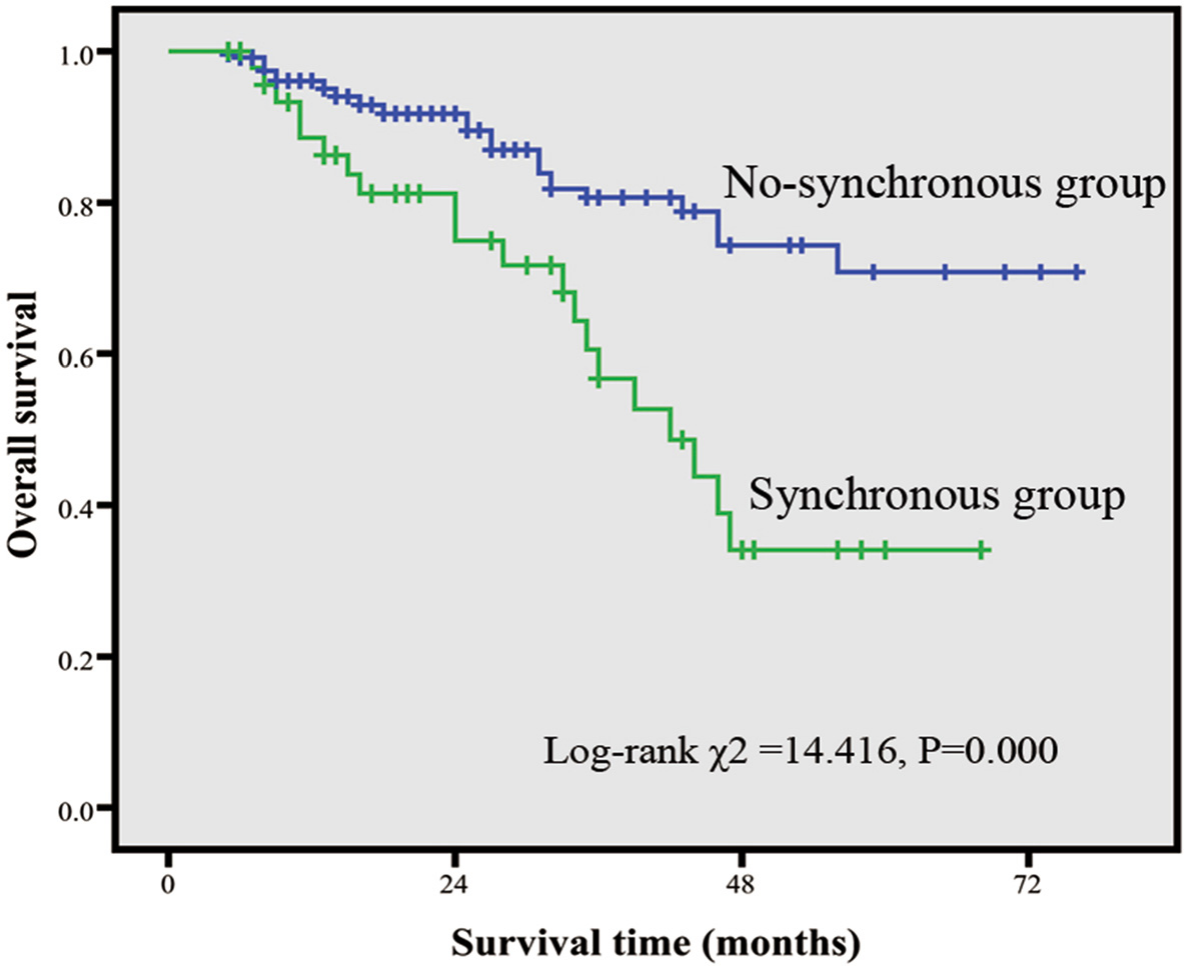
Kaplan-Meier estimates of overall survival (OS) rate. OS was significantly higher in the non-synchronous group than in the synchronous group (*P* < 0.001)

With a median follow-up duration of 32 months (5–76 months), 53 patients died on the last follow-up. In the non-synchronous group, 76 patients presented with GIST-specific progression. The 5-year OS rate was significantly higher in patients with no synchronous digestive tract malignancies than in those with synchronous disease (70.8 vs. 34.1 %, *P* = 0.000; Fig. 2). The median survival rate was not achieved by patients in the non-synchronous group in contrast to 23 months for patients with GISTs synchronous with digestive tract malignancies.

## Discussion

GISTs reportedly occur synchronously with other gastrointestinal neoplasms, including gastric adenocarcinoma and lymphoma, esophageal cancer, colon adenocarcinoma, pancreatic cancer, and hepatocellular carcinoma [9–13]. The most common GIST-associated malignancy is gastrointestinal cancer, which is mainly located in the stomach and then in the esophagus [5, 14]. In our study, 47 out of 286 patients were diagnosed with primary GISTs that are synchronous with primary gastrointestinal neoplasm and the concomitant digestive tumors were in 27 (57.4 %) and 12 (25.5 %) patients which were diagnosed as gastric carcinoma and esophageal carcinoma; this findings were similar to their results. So far, the actual incidence of the coexistence of GISTs and other tumors remains to be determined. Subclinical microscopic gastric GISTs have been reported in the past 3 years. Agaimy et al. found that microscopic gastric GISTs presented in 22.5 % of patients aged 50 years old or older through a series of consecutive autopsies [6]. Kawanowa et al. also reported that microscopic GISTs coexisted with 35 % of resected specimens of gastric carcinomas [4]. With more experience and expertise in the endoscopic examination of the upper gastrointestinal tract, the recognition of incidental subepithelial lesions has significantly increased accordingly. One retrospective study reported the prevalence of subepithelial gastric masses (0.36 %) during routine endoscopy [15]. Results of these studies suggested that GISTs are far more common than previously estimated. Similarly, the synchronous occurrence of GISTs and other primary gastrointestinal tumors may have been underestimated.

According to literature, sporadic GISTs usually arise in the stomach in 40 to 70 % of cases, the small intestine in 20 to 40 % of cases, and the esophagus, colorectum, and other regions of the abdominal cavity, such as the retroperitoneal tissue and mesentery, in less than 10 % of the cases [6, 16]. In the present study, the synchronous GISTs were also most frequently found in the stomach (43 cases, 91.5 %), followed by the small intestine (6.4 %), and rectum (1 case, 2.1 %). Apparently, the most common site of GISTs between synchronous group and non-synchronous group was similar in this study.

In our study, most (95.7 %, *n* = 45) of the synchronous GISTs were accidentally discovered during the surgical exploration of the other gastrointestinal cancer. Only 4.3 % of the coexistent GISTs were identified during preoperative examination. Therefore, a careful search for synchronous GISTs should be carried out during gastrointestinal cancer surgeries. Large, population-based studies have demonstrated that 15–30 % of GISTs are asymptomatic and are usually identified incidentally during surgeries or by postmortem examination [17]. In a study by Miettinen et al., 1765 gastric GISTs were investigated. Results showed that the GISTs were detected incidentally during abdominal surgery or a medical procedure for gallbladder disease in 2.4 % and colorectal carcinomas or adenoma in 1.6 % of the cases [18].

Tumor sizes of GISTs vary in different studies. In the present study, the synchronous GISTs showed a mean size of 1.6 ± 0.4 cm, which was slightly larger than the results from previous investigations. In a study by Agaimy et al., the “microscopic” gastric GISTs in autopsy specimens measured 2–10 mm in size (mean: 5 mm) [6]. Another study reported a median size of 1.5 mm (0.2–4.0 mm) of microscopic GISTs coexisting with gastric adenocarcinoma [4]. Management of GISTs with sizes less than 2 cm is controversial, as the natural history of such neoplasms is unknown. Several retrospective studies demonstrated that the resection of small GISTs presents a favorable oncological outcome. Otani and colleagues resected 35 gastric GISTs (2–5 cm in size) [19]. During the follow-up period (median, 53 months), no local or distant recurrences were found in cases with neoplasms under 4 cm. Moreover, in a retrospective study of 207 patients who underwent gastrectomy or esophagectomy for non-GIST neoplasms, 15 synchronous GISTs in the upper gastrointestinal tract of 11 (5.3 %) patients were found with an average size of 0.5 cm (0.1–4.0 cm) [7]. After a median follow-up of 11 months (2–36 months), no patient experienced GIST recurrence. Synchronous GISTs that were incidentally found during the resection of other gastrointestinal neoplasms may not negatively affect long-term survival, although they often pose very low or low risk of malignant potential. Small GISTs (<2 cm) may be asymptomatic and nonmalignant when diagnosed but have a potential for malignant transformation. In the present study, there were five patients with intermediate/high risk in the non-synchronous group, and they might more likely to experience tumor progression than that of patients with very low/low risk.

Currently, surgical resection is the common treatment for GIST patients. However, a certain risk remains in cases where only the primary gastrointestinal tumors are resected completely. The residual GISTs may grow and ulcerate, cause obstruction and bleeding, and pose difficulties to the postoperative evaluation of the primary gastrointestinal neoplasms. Therefore, R0 resection should be achieved in all surgical procedures for GISTs. Small GISTs can be safely performed by means of endoscopic resection, as reported by Shen et al. [20]. As GISTs rarely (about 5 %) metastasize to lymph nodes, routine lymph node dissection around GIST is often unnecessary. The present data showed that the OS rate was significantly higher in patients without digestive tract malignancies. This finding is consistent with those of a previous report [5].

Multiple studies showed that among patients with primary GISTs, 14–27 % have synchronous gastrointestinal neoplasms. By contrast, only 3–5 % of the general population may have the gastrointestinal tumors, which reveals a conspicuous discrepancy between the two groups. Currently, various hypotheses attempt to explain the synchronous existence of GISTs with other gastrointestinal tumors. These hypotheses include the relations to genetic predisposition, environmental risk factors, mutagenic effect from previous radiation or chemotherapy, *Helicobacter pylori* infection, chronic atrophic gastritis, and coincidental findings [21–24]. Whether the synchronicity is a simple incidental association or a legitimate causal relationship between the occurrences of the two tumors is still unknown. Tada et al. [23] believed that a stomach harboring a leiomyosarcoma may have a tendency to develop malignant epithelial lesions. On the basis of accumulated reports, Maiorana et al. [24] proposed that in cases of synchronicity, a single carcinogenic agent may have interacted with two neighboring tissues and induced the development of tumors of different origins in the same organ, such as the gene mutation of KIT or PDGFRA. However, Ponti et al. [25] speculated that a small subset of GISTs that are negative for the KIT- and PDGFRA-activating mutations may be inherited and may occur as part of a multi-neoplastic disease. Some researchers believe in the existence of a certain carcinogen that can act on different tissue cells in the same or adjacent organs and can result in two types of tissue differentiation. Other hypotheses, such as that related to *H. pylori* infection, still lack identifiable experimental evidence. However, some researchers reported that there was no relationship existing between the synchronous occurrence of these two tumors and this phenomenon is only coincidental [26].

## Conclusions

In conclusion, the synchronous occurrence of GISTs and other gastrointestinal tumors is more common than previously estimated. Surgeons should be vigilant in recognizing a coexisting tumor before or during surgery and be prepared to modify the surgical plan accordingly. Patients with GISTs synchronous with other gastrointestinal cancers showed worse prognoses than those with non-synchronous disease. Further studies are required to elucidate the exact molecular and genetic mechanisms underlying the carcinogenesis and progression associating GISTs with synchronous tumors.

## Abbreviations

ECOG: Eastern Cooperative Oncology Group
GIST: gastrointestinal stromal tumors
IM: imatinib mesylate
NIH: National Institutes of Health
OS: overall survival
